# ACSNI: An unsupervised machine-learning tool for prediction of tissue-specific pathway components using gene expression profiles

**DOI:** 10.1016/j.patter.2021.100270

**Published:** 2021-06-11

**Authors:** Chinedu Anthony Anene, Faraz Khan, Findlay Bewicke-Copley, Eleni Maniati, Jun Wang

**Affiliations:** 1 Centre for Cancer Genomics and Computational Biology, Barts Cancer Institute, Queen Mary University of London, London EC1M 6BQ, UK

**Keywords:** pathways, systems biology, neural network, cell signaling, gene-regulatory networks, machine learning, dimension reduction, gene expression, autoencoder

## Abstract

Determining the tissue- and disease-specific circuit of biological pathways remains a fundamental goal of molecular biology. Many components of these biological pathways still remain unknown, hindering the full and accurate characterization of biological processes of interest. Here we describe ACSNI, an algorithm that combines prior knowledge of biological processes with a deep neural network to effectively decompose gene expression profiles (GEPs) into multi-variable pathway activities and identify unknown pathway components. Experiments on public GEP data show that ACSNI predicts cogent components of mTOR, ATF2, and HOTAIRM1 signaling that recapitulate regulatory information from genetic perturbation and transcription factor binding datasets. Our framework provides a fast and easy-to-use method to identify components of signaling pathways as a tool for molecular mechanism discovery and to prioritize genes for designing future targeted experiments (https://github.com/caanene1/ACSNI).

## Highlights

• ACSNI uses deep neural network to decompose transcriptomic measurements
• ACSNI maps pathway components to target biological or clinical phenotypes
• ACSNI robustly extracts regulatory output of transcription factors
• ACSNI enables annotation of genes with unknown functions

## The bigger picture

Methods that group genes into functional units to quantify pathway activities are critical in the analysis of biological systems. Although many components of biological pathways have been described in detail, these tend to be limited to well-studied genes. In contrast, the majority of possible components remain unexplored. Here, we present a machine-learning tool for constructing and predicting tissue-specific components of biological pathways from large biological datasets. Our algorithm, ACSNI, can tackle incomplete pathway descriptions and enhance current pathway analysis methods' performance. We anticipate that, by dissecting the complex signals in biological data in a flexible and context-specific manner, ACSNI can facilitate the full characterization of physiological systems of interest.

## Graphical abstract

**Figure:**
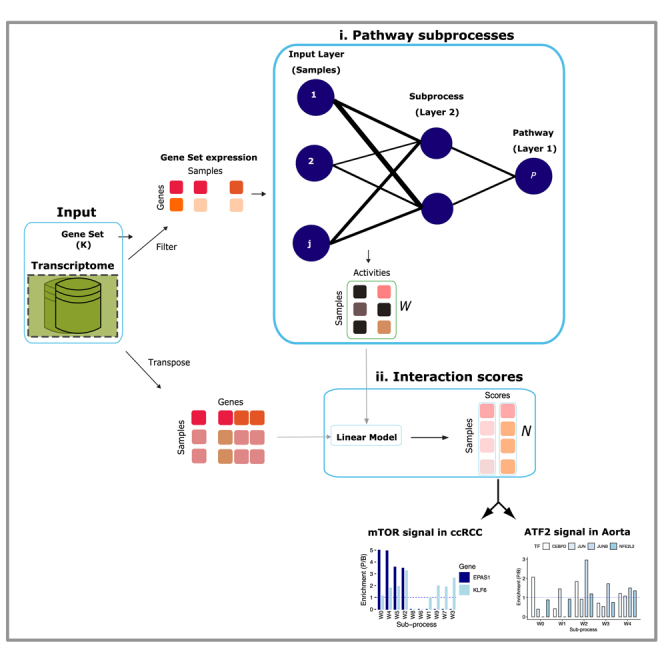

## Teaser

ACSNI software presents a flexible approach to discover unknown components of a biological pathway. We demonstrate through simulation and biological user cases that ASCNI has superior performance in inferring pathway activities compared to other approaches. In particular, it is able to identify unknown components of pathways in a tissue-specific manner, facilitating the full characterization of physiological systems of interest.

## Introduction

One feature common to all cells is the dynamic ability to coordinate activities through many pathways that receive and process signals from the environment and different cell regions.^1^ Hence, there has been a persistent interest in developing pathway analysis approaches that group genes into functional units and elucidate molecular mechanisms. Most approaches, especially the techniques of pathway enrichment analysis and network-based modeling, require accurate and comprehensive pathway descriptions with annotated regulatory components (genes). However, the identities of the components within these biological pathways and their regulatory interactions remain either wholly unknown or partially understood.^2^ Even among well-studied pathways, detailed annotations are scarce for their disease- and tissue-specific components. This unmet need argues for an expanded effort to identify these unknown components.

Genetic, biochemical, and biophysical techniques such as small interfering RNAs, small molecular inhibitors, immune precipitation, and gel filtration are common procedures for identifying molecular functions and pathway components.^3^, ^4^, ^5^ The first step toward designing these experiments is the identification of candidate genes and their interacting partners. The large volume of public gene expression profile (GEP) datasets, such as the tissue RNA expression profiles in the Genotype-Tissue Expression (GTEx)^6^ and The Cancer Genome Atlas (TCGA)^7^ projects, allow for initial inference of pathway circuits and prediction of their unknown components in a tissue- and disease-specific manner. One popular approach applied to these large datasets involves using pairwise correlation to represent the relationships between genes.^8^ Visualizing these interactions as a network annotated with external functional information such as STRING^9^ and GeneMANIA^10^ reveals interactions of known pathway genes and helps to discover additional related genes. However, these approaches make assumptions that do not account for the multivariate nature of gene regulation.^2^ For example, the transcription of a single gene proceeds through the binding of transcription factors on enhancer regions and the subsequent recruitment of many co-activators and complexes that modify chromatin structures and promote the assembly of the basal transcriptional machinery.^11^ These complexities are not easily captured by simple pairwise analysis between genes and the resultant binary interaction networks.

Another approach for identifying unknown components of pathways involves first estimating a summarized pathway activity from GEPs based on prior knowledge of the target pathway using gene set enrichment analysis tools, such as gene set variation analysis (GSVA), pathway-level analysis of gene expression (PLAGE), single-sample gene set enrichment analysis (ssGSEA), and the combined *Z* score (*Z* score),^12^, ^13^, ^14^, ^15^ then correlating this compact value with the rest of the genes to identify new components of the target pathway. However, genes function in more than one pathway, and pathways contain subprocesses,^1^ meaning that a single variable representation of a pathway is unlikely to capture the target pathway's dynamics.

Here we provide software termed automatic context-specific network inference (ACSNI), which leverages artificial intelligence for the reconstruction of a biological pathway and aids the discovery of pathway components and classification of the crosstalk between pathways in specific tissues. The method draws on the principle that cell signaling networks are organized into several small, highly connected modules (herein called subprocesses) that combine hierarchically into larger units to regulate cellular functions.^16^ ACSNI combines the widely used and general-purpose open-source TensorFlow-Keras library with a probabilistic ensemble approach in a manner that adapts to the particular needs of systems biology projects. For example, it has functions that enable flexible adjustment for gene weights within the pathway and linking pathway subprocesses to a phenotype. We demonstrate the utility of ACSNI through three different use cases, including the investigation of the mTOR signaling pathway in kidney tumor and normal samples, exploring the ATF2 network in the aorta, and identification of HOXA transcript antisense RNA myeloid-specific 1 (HOTAIRM1) target genes in kidney tissues. We find that ACSNI generates validated and detailed, tissue-specific pathway circuitry and components. The ACSNI predictions can guide researchers in designing targeted experiments to study the molecular mechanisms that underlie a biological state.

## Results

### Overview of the ACSNI method

**Figure 1:**
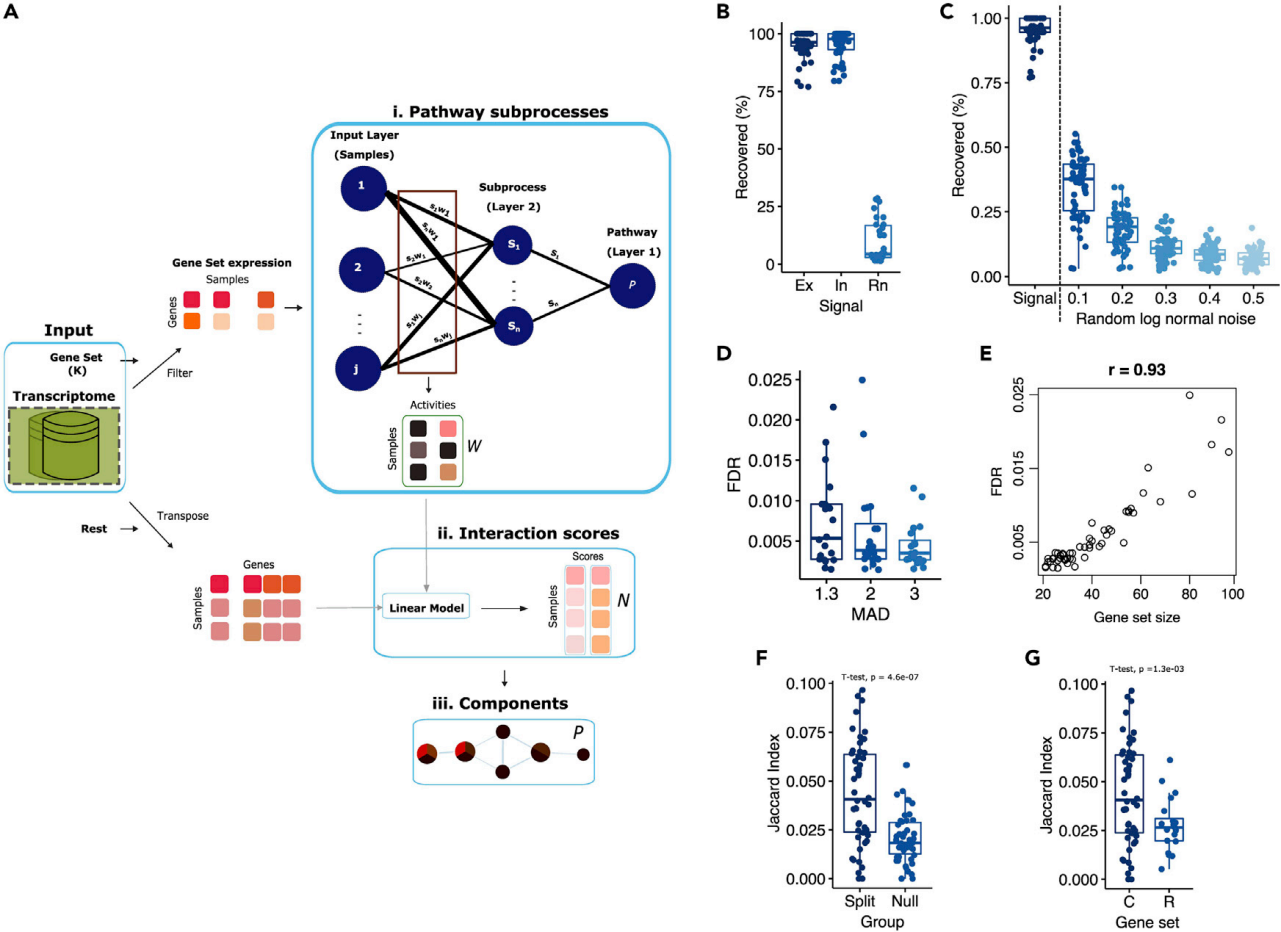
Overview of the ACSNI method and the robust signal reconstruction capabilities (A) ACSNI reduces the expression of a gene set into a small number of subprocesses and derives corresponding gene interactions, while constraining the optimization to reduce technical noise. Given two inputs, the expression matrix, and the binary gene set membership (pathway representation), the algorithm starts by splitting the transcriptome into two parts: (1) expression of the genes in the gene set and (2) the expression of the rest of the transcriptome (transposed). It then extracts the subprocess activities across samples (step i: W) from the expression profiles of the gene sets, interacts the subprocesses with the expression of the rest of the transcriptome to extract subprocess-gene interaction scores (step ii: N), and classifies the scores to infer extended network of the pathway represented by the gene set (step iii: P). (B) Box plot of the percentage of the simulated signal recovered from the analysis of 50 gene sets against five GTEx tissue expression samples. Ex represents the gene set signal, In the expected signal, and Rn the random signal. (C) Box plot depicting the effect of random noise (log normal) on the percentage of gene set signal recovered from the analysis of 50 gene sets against five GTEx tissue expression samples. (D) Box plot showing the false discovery rates (FDR) from the analysis of 50 gene sets against five GTEx tissue expression samples (estimated as the number of genes from the random signal divide the total number of predicted genes in B). (E) Scatterplot of the relationship between FDR and size of the gene set. (F) Box plot of Jaccard index of the similarity of predicted genes between two independent split or with one shuffled split (Null) across 50 curated gene sets. (G) Box plot comparing the Jaccard index of predicted genes between two independent expression splits for randomly generated (R, n = 20) and curated (C, n = 50) gene sets. See also Figures S1 and S4.

ACSNI is a three-step method to infer tissue-specific components (genes) of a pathway from tissue GEPs. It performs (1) unsupervised derivation of pathway subprocesses, (2) estimation of subprocess-gene interaction scores, and (3) inference of tissue-specific components of the target pathway (Figure 1A; see experimental procedures). ACSNI requires two input datasets: a matrix of expression values, with genes listed in rows and tissue samples listed in columns, and a file detailing gene set membership, representing prior knowledge of gene functions, such as a gene set from the Pathway Interaction Database (PID)^17^ and the MSigDB database.^18^ Depending on the research question, ACSNI can take an additional file containing weights (as integer values) for the genes in the second input. These data may represent transcription factor (TF) activity level or kinase function. In the first step of ACSNI, we derive multivariate pathway representation that defines the activity of the subprocesses in each sample (step i, Figure 1A). The step is performed with a deep neural network (DNN) that decomposes the expression of the gene set into pathway subprocesses under the assumption that genes function in multiple subprocesses. In the second step, the subprocess-gene interaction analysis is conducted on the whole transcriptome using a linear model. We extract the model's coefficient of determination as subprocess-gene interaction scores (step ii, Figure 1A). We then classify the interaction scores to identify the tissue-specific components of the pathway (step iii, Figure 1A). The final output of ACSNI can be further mined to identify biologically relevant pathway subprocesses and components.

### Performance evaluation

To assess the performance of ACSNI in reconstructing signaling networks and their subprocesses, we utilized pseudo-simulated and real expression datasets. It is natural to ask to what extent the ACSNI predictions improve on current methods for inferring the component of a pathway from GEPs. Unfortunately, there are no existing tools that provide such a granular view of pathway subprocesses. Therefore, we focused our performance evaluations on reconstruction of simulated signals and concordance between two cohorts of similar samples.

#### ACSNI has robust signal reconstruction capabilities with low type I error rate

In biological systems (i.e., tissues or organs), multiple signaling pathways are executed concurrently with a certain level of dispersion (i.e., a measure of pathway activity) across a group of samples. ACSNI has been designed to extract differences in pathway activity in a cohort of samples and detect unknown components of the pathway. To assess how well the algorithm achieves this aim, we first evaluated its ability to reconstruct a known signal using pseudo-simulated GEPs under different parameter settings (see experimental procedures). We simulated random GEPs with specific expected signals and noise signals, as described in datasets for simulation studies. Then, for each GEP and the corresponding gene set, we tested the null hypothesis for no difference in the proportion of the predicted genes overlapping the expected or random signals. To examine the effects of different ACSNI parameters, we ran the analysis under different parameter settings of classification threshold (Alpha: 0.01, 0.05), latent space dimension (Percentage: 5, 10, 15, 20, 25, 30), feature selection threshold (Median absolute deviation: 1.2, 1.3, 3), and regularization (yes, no), repeating the analysis 50 times with three ensemble models each to obtain more stable results (see experimental procedures). Testing for the overlap between the predictions and the expected signals revealed that ACSNI recovered most of the expected signals (average percentage of overlap = 98%, n = 250, Figure 1B) compared with 3.5% random overlaps. The concordance of ACSNI predictions with the expected signals is due to the robustness of the DNN extraction core that dissects the gene set expression patterns into subprocesses (see experimental procedures). As expected, ASCNI's ability to recover the expected signal is negatively associated with the level of technical noise (Figures 1C and S1A, see experimental procedures). These results suggest that the quality of the prediction is dependent on the level of technical noise (examples, errors due to RNA quality, fragmentation, and amount of input) in the GEP measurements. On average, more than 30% or 45% of the expected signal was recoverable at 10% and 5% noise, respectively (Figures 1C and S1A). Previous studies indicated that in RNA-sequencing (RNA-seq) data sequenced to sufficient depth, the level of technical noise is generally lower than 10%, while the rest of the expression variation is explained by biology and experimental conditions.^19^^,^^20^ Thus, we believe that ACSNI retains its predictive power when analyzing current real expression datasets across various experiments and conditions (noise level <10%). Note that latent space dimension, classification threshold, and feature selection threshold had no effect on ACSNI's ability to recover expected signals.

To measure the type I error rate, we divided the number of random predictions (false positives) by the total number of predictions (total positives) in the above simulation and assessed the impact of different parameter settings. We observed a median false discovery rate (FDR) of 0.0045 (range, 0.003–0.005) for default parameter settings across three feature selection thresholds (Figure 1D; see experimental procedures). This observation indicates that ACSNI has a low (<1%) rate of false predictions. Applying a conservative feature selection threshold (i.e., genes with median absolute deviation greater than 3 across the cohort) before running ACSNI resulted in tighter control of the FDR (median = 0.0038; range, 0–0.01) (Figure 1D). We next tested how key parameters, including classification threshold, dimension of the latent space, and gene set size, affected the overall type I error rate of ACSNI. Relaxing the classification threshold 0.01 and 0.05 significantly increased the FDR (Wilcoxon test p = 3.4 × 10^−13^, Figure S1B). Although regularization did not affect the FDR (Figure S1C), increasing the dimension of the latent space (a percentage of the gene set size) significantly increased the FDR, but using 5% made the FDR unstable (Figure S1E). We found a positive correlation (Pearson's r = 0.93) between gene set size and FDR (Figure 1E). These associations are expected because larger gene sets and genes with a low variability are more likely to result in aberrant predictions. However, even in the case of a larger gene set (>80, Figure 1E) we expect the FDR to be less than 1% (see experimental procedures).

We then tested whether the approach was a robust predictor of pathway components across different cohorts of similar samples. To this end, we randomly sampled two non-overlapping groups of 290 samples from the 580 samples of the GTEx healthy adult lung dataset to generate a training cohort and a test cohort. We also randomly shuffled the test cohort to generate a null model (see experimental procedures). Next, we individually analyzed these three cohorts (training, test, and null model cohorts) with ACSNI using 50 gene sets from the MSigDB database^18^ and quantified the similarity of the inferred circuitry between the training cohort and the test cohort compared with the similarity between the training cohort and the null model. Predicted circuitry from the training cohort was significantly more similar to the test cohort compared with the null model (t test, p = 4.6 × 10^−7^, Figure 1F). We repeated the above analysis using 20 randomly assigned gene sets and observed no difference between the real cohorts and the null model, as the random inputs lacked underlying regulatory mechanisms. Consistently, manually curated gene sets show a significantly higher similarity between training cohort and test cohort compared with the simulated gene sets (t test, p = 1.3 × 10^−3^, Figure 1G). The level of similarity between two expression cohorts is independent of the type of pathway and the size of the gene set (Figure S2A). Combined, these results indicate that ACSNI predictions are reproducible, with datasets of similar biological and regulatory information.

#### ACSNI identified the mTOR crosstalk with KLF6 and EPAS1 signals in ccRCC

To evaluate the performance of ACSNI on real data, we simulated a condition whereby known context-dependent signaling crosstalk was expected. Specifically, a recurrent event in clear-cell renal cell carcinoma (ccRCC) is the hyperactivation of the mTOR signaling that promotes oncogenic metabolic programs.^21^^,^^22^ The crosstalk between mTOR, KLF6, and EPAS1 (HIF2A) signaling contributes to this metabolic program that drives ccRCC progression. KLF6 is a zinc finger DNA-binding TF activator of mTOR signaling that co-regulates lipid metabolism, cell growth, and cell fate in ccRCC.^23^ mTOR regulates transcription of EPAS1,^24^ which in turn activates a superenhancer that supports KLF6 expression,^23^ creating a complex feedback loop between mTOR, KLF6 and EPAS1 signaling.

**Figure 2:**
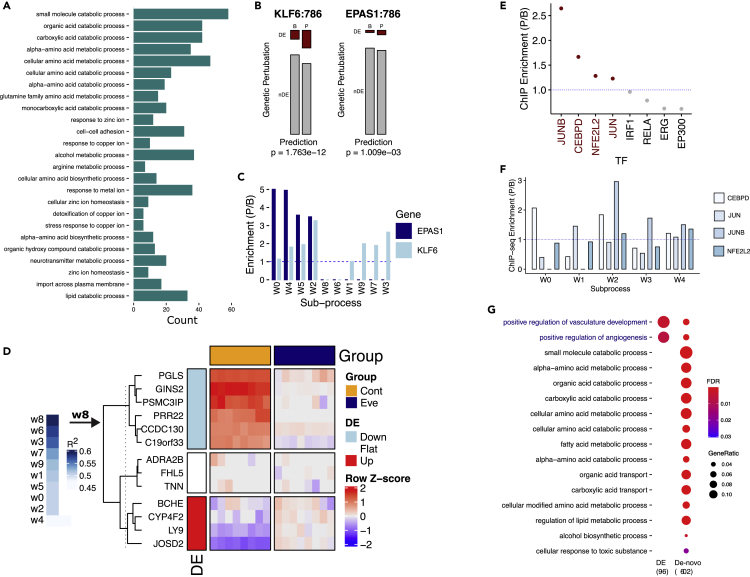
ACSNI identified pathway components and signaling crosstalk (A and B) (A) Bar plot of the top 25 significantly enriched biological processes associated with the predicted 1,166 genes mTOR signaling components. Bars are ordered from top to bottom according to FDR values. (B) Association of ACSNI-predicted mTOR signaling genes (TCGA) with DE genes in KLF6 and EPAS1 knockout in ccRCC cells, divided into datasets (KLF6: 786 = GEO: GSE115763, EPAS1: 786 = GEO: GSE115389). Chi-squared tests (Chisq, p) and ACSNI-predicted (P) and background (B) are indicated on top of the panel. DE and nDE represent differentially or non-differentially expressed genes at adjusted p value of <0.05, respectively. (C) Bar plot comparing enrichment of DE genes from EPAS1-KO (red) and KLF6-KO (blue) across the different ACSNI-derived subprocesses of mTOR signaling (TCGA). (D) (Left) Heatmap of the coefficient of determination (R^2^) of the linear model of ACSNI-derived mTOR activity and disease status (cancer or normal adjacent tissues) across ten subprocesses. The higher the R^2^, the more significantly related is the subprocess to disease status. (Right) Expression of mTOR subprocess *w8*-associated genes in ccRCC cell lines following the inhibition of mTOR pathway with everolimus (Eve) compared with vehicle control (Cont) (GEO: GSE106819). Within the plot differential expressions at FDR < 0.05 (DE) are indicated (upregulated, red; downregulated, blue; unchanged, white). (E) Ranked dot plot of the ratio of transcription factor (TF) ChIP density at the promoter regions (± 1 kb from TSS) of the ACSNI-predicted ATF2 signaling genes relative to background genes in artery aorta. DNA-binding domains are highlighted in red. (F) Bar plot comparing ratio of TF ChIP density at the promoter regions (±1 kb from TSS) of the ACSNI-predicted ATF2 signaling genes across the different ACSNI-derived subprocesses from artery aorta. (G) Gene ontology analysis of biological processes associated with predicted HOTAIRM1 genes and DE genes in HOTAIRM1. See also Figures S2–S4.

Consistent with the above, we hypothesized that mTOR signaling network derived from GEPs in ccRCC patients could enrich for both KLF6 and EPAS1 signals. To test this hypothesis, we collected data of 73 ccRCC samples with matched normal adjacent tissues from the TCGA ccRCC project (KIRC, n = 146). We applied ACSNI to the RNA-seq data using a publicly available curated mTOR signaling gene set from the PID (n = 67).^17^ We derived ten subprocesses of mTOR signaling that were strongly associated with 1,166 genes (Figure S2B). We next evaluated the biological processes associated with mTOR signaling in this context. Using gene ontology analysis, we found a strong enrichment of catabolic processes and amino acid biosynthesis ontologies (Figure 2A), consistent with the role of mTOR signaling in cell metabolisms.^25^ To assess whether the ACSNI-predicted mTOR signaling captured KLF and EPAS1 signal, we first defined KLF6 and EPAS1 signals as differentially expressed (DE) genes from publicly available gene perturbation datasets: (1) KLF6 knockout (KLF6-KO, DE = 1,378 genes) and (2) EPAS1 knockout (EPAS1-KO, DE = 303 genes) in the 786 ccRCC cell line.^23^^,^^26^ The inferred network had significant over-representation of KLF6 (1.47-fold, chi-squared p = 1.763 × 10^−12^, Figure 2B) and EPAS1 (2.07-fold, chi-squared p = 1.009 × 10^−3^, Figure 2B) signals compared with background enrichment (i.e., all genes in the expression dataset). Inspecting the ACSNI subprocesses and their associated genes, we found unique associations with KLF6 and EPAS1 signals (Figure 2C). Subprocesses, *w3*, *w7*, and *w9* were exclusive to KLF6 signal and *w0* was exclusive to EPAS1, while *w4*, *w5*, and *w2* captured both signals but have relatively more enrichment of EPAS1 signal (Figure 2C). Three subprocesses (*w8*, *w6*, and *w1*) appear to capture signals unrelated to KLF6 and EPAS1. These observations demonstrate that ACSNI models the complexities of a signaling network as represented by an experimentally curated gene set.

We next investigated the specificity of ACSNI by comparing our predictions with KLF6 and EPAS1 signals from non-ccRCC cells lines as non-specific controls (including endothelial cells [human umbilical vein endothelial cells—HUVECs] and primary peripheral blood mononuclear cells [PB-CD34^+^]). We found an insignificant over-representation for DE genes (n = 119) derived from EPAS1-KO in HUVECs (0.91-fold, chi-squared p = 7.482 × 10^−1^, Figure S2C), underlining the advantage of detecting context-specific signaling crosstalk. We observed a significant enrichment for DE genes (n = 8,487) derived from KLF6-KO in PB-CD34^+^ (1.2-fold, chi-squared p = 1.56 × 10^−9^, Figure S2C), suggesting that the mTOR-KLF6 axis may be applicable to multiple tissues. However, these data had an unusually high level of DE genes (60%, 8,487 out of 14,260), which may be related to the fact that the experiment was performed in primary cells. To further validate our predictions, we applied ACSNI to an independent dataset of GEPs of tumor and normal adjacent tissues from ccRCC patients (n = 22, GEO: GSE102101)^27^ using the same mTOR gene set described above. We evaluated the robustness of the initial predictions against the new predictions using a randomization test (see experimental procedures). We found that 19% (98/516, only genes measured in both datasets) of the predicted genes among these data overlap with the KIRC predictions (Figure S3A, left panel), which is significantly higher than would be expected by random chance (empirical p = 0.0, 95% confidence interval 23–44) (Figure S3A, right panel). There was no enrichment of KLF6 and EPAS1 signals in the corresponding null models (Figure S3C; see experimental procedures), confirming that our predictions and validation were robust. As an additional layer of performance evaluation, we next compared ACSNI global predictions against predictions based on pathway activities estimated from GSVA, PLAGE, ssGSEA, and *Z* score (see experimental procedures).^12^, ^13^, ^14^, ^15^ ACSNI recovered the strongest enrichment of KLF6 (1.91 mean fold enrichment) and EPAS1 (2.17 mean fold enrichment) signaling crosstalk with mTOR pathway compared with mean fold enrichment of 1.71 (KLF6) and 1.44 (EPAS1), 1.17 (KLF6) and 1.16 (EPAS1), 0.77 (KLF6) and 0.55 (EPAS1), and 0.94 (KLF6) and 0.89 (EPAS1) mean fold enrichment for GSVA, PLAGE, ssGSEA, and *Z* score, respectively (Figure S3B). These results indicate that our framework can provide additional context-specific information to aid the interpretation of GEPs.

#### ACSNI-derived mTOR subprocesses predict clinical traits in ccRCC

The identification of biologically or clinically essential pathway subprocesses is a crucial aim of many signaling network analyses. ACSNI provides additional functions to map the derived network to a target biological or clinical variables. To demonstrate the utility of this function, we analyzed the distribution of the mTOR subprocesses across the tumor and normal adjacent tissue from the TCGA datasets (variable: disease status). We found that three subprocesses (*w8*, *w6*, and *w3*) were strongly associated with the disease status (Figure 2D, left panel). Interestingly, two of these (*w8* and *w6*) were not associated with KLF6 nor EPAS1 signal (Figure 2C), suggesting that they may harbor new insight into mTOR-dependent mechanisms of ccRCC development. To investigate this hypothesis, we first sought to confirm the regulation of the *w8-*associated genes (the top disease-associated subprocesses) by mTOR. To this end, we analyzed published GEP data (GEO: GSE106819)^28^ which utilized the mTOR inhibitor, everolimus, to characterize the differential gene expression changes that occur due to mTOR signaling in ccRCC cell lines. Among the 13 genes associated with *w8* in ACSNI predictions, 77% (10/13 genes) were significantly altered by mTOR inhibition (Figure 2D, right panel), confirming that *w8* is within the mTOR signaling network. Of the total ten *w8* genes altered by everolimus, 70% (GINS2, CCDC130, PRR22, CYP4F2, PGLS, LY9, and BCHE) have been identified as essential ccRCC genes in a CRISPR genome-wide loss-of-function screen^29^ (Figure S2D). These observations suggest that these genes may co-regulate oncogenic programs in ccRCC through mTOR signaling.

#### ACSNI identified ATF2-dependent targets of bZIP transcription factors in artery aorta

Identification of functional TFs and their regulatory output is essential for understanding gene-regulatory mechanisms in many cellular processes.^30^ ACSNI can provide information about the context-specific regulatory output of TFs in a gene set. As an example, here we focus on cAMP response element-binding proteins (CREBs) and CREB-like TFs that dominate ATF2 signaling (10 out of 13 TFs). These genes represent a large family of TFs that bind cAMP-responsive elements and share a basic leucine zipper DNA-binding (bZIP) domain. Complex dimerization between these TFs increases the selectivity of bZIP-DNA interactions and binding diversity, allowing them to regulate multiple vascular functions.^31^ We hypothesized that ATF2 network circuitry derived from GEPs of artery aortas should enrich targets of the bZIP TFs.

We analyzed the ATF2 TF signaling gene set from the PID (n = 31)^17^ in artery aorta (n = 433) GEPs from the GTEx project.^6^ We produced four subprocesses associated with 683 genes, representing the context-specific activity of bZIP TFs (Figure S2E). Using an independent dataset of human TF chromatin immunoprecipitation (ChIP)-sequencing measurements from primary human aortic endothelial cell lines from chip-atlas (https://chip-atlas.org), we evaluated the enrichment of the available eight TFs (bZIP = 4, others = 4) at the promoter regions (±1 kb from transcription start site [TSS]) of the predicted genes compared with the background (enrichment = mean peak density of predicted genes/mean peak density of background genes). ACSNI-predicted genes were strongly enriched for bZIP transcription factor binding (ratio > 1) but depleted of other families of TFs (ratio < 1) (Figure 2E). We observed no significant enrichment of bZIP TF for predictions from a null model derived by random shuffling of the expression matrix (Figure S3E; see experimental procedures), confirming that our predictions and validation were robust. On average, the predicted genes had significantly higher binding of JUNB (Wilcoxon test, p = 2.083 × 10^−9^), CEBPD (p = 1.278 × 10^−4^), NFE2L2 (p = 1.168 × 10^−3^), and JUN (p = 1.041 × 10^−2^) compared with the background genes. Conversely, we observed significantly lower binding for EP300 (p = 7.582 × 10^−32^), RELA (p = 7.132 × 10^−10^), ERG (p = 1.841 × 10^−5^), and IRF1 (p = 3.148 × 10^−2^) compared with the background genes. Interrogating the ACSNI-derived subprocesses of ATF2 signal and their associated genes revealed unique associations with different bZIP dimerization patterns (Figure 2F). Specifically, *w0*, *w1*, and *w3* were exclusive to CEBPD, JUN, and JUNB, respectively. While *w2* enriched for NFE2L2, JUNB, and CEBPD, with higher JUNB, *w4* enriched for all four bZIP TFs measured in these data (Figure 2F). These observations suggest that ACSNI can segregate different TF signals into specific subprocesses of the *W* matrix by leveraging external knowledge to anchor RNA expression to TF activities underlying complex cell processes.

To benchmark our results, we analyzed the enrichment of these TF signals on the genes predicted based on pathway activities estimated by GSVA, PLAGE, ssGSEA, and *Z* score (see experimental procedures). Predictions derived from pathway activity scores generated from these methods showed high enrichment rank for non-bZIP TFs (EP300, RELA, ERG, and IRF1) and largely low in signal for the bZIP TFs (JUNB, CEBPD, NFE2L2, and JUN) (Figure S3D). Compared with ACSNI, the alternative approach based on these methods showed non-significant enrichment for all the TFs regardless of the DNA-binding domain. These results demonstrate how ACSNI predicts context-specific TF targets and enables discovery of pathway-level regulatory mechanism and interpretation of expression profiles.

#### ACSNI annotates the function of HOTAIRM1 using *de novo* derivation of gene sets

ACSNI also provides tools to help annotate genes with unknown functions, such as long non-coding RNAs (lncRNAs). To demonstrate this utility, we focused on the annotation of the lncRNA HOTAIRM1. Specifically, HOTAIRM1 is a natural antisense transcript of the HOXA1 gene that plays roles in kidney differentiation and regulation of HIF1-dependent angiogenic pathways.^32^ We hypothesized that the ACSNI-inferred network of HOTAIRM1 could reveal its kidney-specific functions.

To this end, we leveraged the GTEx project of GEPs in normal kidney tissues and investigated the network of HOTAIRM1. Because this case deals with a single gene, we utilized the *ACSNI-derive* module to first generate a *de novo* gene set (n = 50) before running two iterations of ACSNI (see experimental procedures). We identified 729 genes that were strongly associated with the network of HOTAIRM1 in kidney. To evaluate the functional relevance of the predictions, we initially analyzed published data (GEO: GSE136604)^32^ that utilized knockdown of HOTAIRM1 to characterize the differential gene expression changes that occur due to its activity in human kidney cells (HOTAIRM1-KO). We then cross-referenced the ACSNI-predicted genes to establish whether the predictions enriched the lncRNA activity compared with random backgrounds. For the genes measured in both datasets (n = 390), the DE genes identified in the HOTAIRM1-KO data (n = 85) showed a significant overlap with ACSNI-predicted HOTAIRM1 genes (12/85, 14.11%; empirical p = 8 × 10^−5^, only genes measured in both datasets, Figure S3F). To assess the biological processes regulated by HOTAIRM1 in this context, we performed gene ontology analysis on the ACSNI-predicted genes compared with the ontologies associated with the DE genes in the HOTAIRM1-KO. The results showed strong concordance in the enrichment of ontologies involved in vascular functions and angiogenesis (Figure 2G). We also observed an enrichment of metabolic processes, suggesting that HOTAIRM1 may play a role in cell metabolism in the kidney (Figure 2G).

We next evaluated the robustness of the predictions by comparing the enrichment with predictions from a null model derived by random shuffling of the expression matrix. We found no significant enrichment of genes differentially expressed in the HOTAIRM1-KO data (Figure S2F), confirming that our predictions and validation using the non-shuffled data did not occur by chance. Correlation and differential gene expression analysis are the two commonly used methods for identifying functional lncRNA targets from GEPs. Thus, we compared ACSNI predictions with direct correlation or differential expression test for pairwise gene interaction analysis on the same input expression data above (see experimental procedures). ACSNI predictions recovered the most overlap with HOTAIRM1 signal (14.11%) compared with zero overlap and 1.19% overlap for differential expression-based and correlation approaches, respectively (Figure S3G). Consistently, genes predicted by both methods were not enriched in ontologies involved in vascular functions and angiogenesis. These results suggest that ACSNI can leverage the annotation of protein coding genes to provide functional annotation for poorly annotated genes.

## Discussion

Our knowledge of biological pathways and their components is far from complete. This situation limits the accuracy of the current pathway and network analysis methods that depend on accurate and detailed pathway annotations. ACSNI enables the identification of tissue-specific components of biological pathways from gene expression data. This approach overcomes the limitations of using composite scores or representative values, which are unable to fully capture the process or handle the inherent variability in GEPs within a gene set. By contrast, ACSNI can capture pathway modules while reducing the impact of technical noise. This capability was demonstrated in our identification and validation of mTOR signaling crosstalk with KLF6 and EPAS1 in ccRCC. ACSNI can also reveal the context-specific output of TFs in a gene set and identify lncRNA network in expression data. This utility is especially vital in mechanistic studies where the target is context-specific gene regulation. By using prior knowledge and interrogating associated expression variability in GEPs, ACSNI can identify biological network within a specific tissue and has the flexibility to apply weights to the prior information.

Using an autoencoder the core of ACSNI decomposes GEP into multiple pathway subprocesses, which are then used to infer unknown pathway components. Other dimension-reduction methods, including principal component analysis (PCA) and non-negative matrix factorization (NMF), could also theoretically be used to estimate pathway subprocesses from GEPs. The key limitation of these methods is that the mapping from the gene expression space to the low-dimensional representation is restricted to be linear.^33^ However, an accurate model of the activity of biological pathways encoded in GEPs requires a mix of both linear and non-linear transformations. Our comparative analysis showed that replacing the autoencoder in step 1 with PCA or NMF resulted in no enrichment of the expected signal (Figure S4C) or extraction of non-specific signals (Figure S4D). Therefore, the autoencoder approach used for step 1 is more capable of extracting biologically meaningful results compared with simple linear reductions (Figures S4C and S4D), consistent with previous studies.^34^

One potential limitation is a lack of prior knowledge in the form of a gene set; this can be partially remedied by *ACSNI-derive*, which enables *de novo* generation of gene sets as demonstrated in the annotation of HOTAIRM1 functions. In addition to the availability of prior information, ambiguous annotations, sample size, gene set size, and expression variance can all affect the performance of ACSNI. Currently, ACSNI depends on RNA expression across a cohort of samples to infer pathway circuitry and will miss post-translation interactions, since RNA levels may not correlate with protein output.^35^ Despite these limitations, ACSNI supplements the existing methods to identify pathway components and extract biological meaning from transcriptomic data and can be applied to study processes that have little prior knowledge.

Independent validation of the inferred network is an essential step in the discovery of pathway components and their functional interactions. Ideally, such analysis should involve independent genetic perturbation data, reporter assays, or DNA occupancy analyses as demonstrated in our use cases. However, genetic perturbation or DNA occupancy data may not always be available. Literature mining can be an alternative approach, but the results may be confounded by publication bias. Our results show that this framework effectively infers biological networks from GEP datasets and enables the discovery of novel functional interactions. Analysis of publicly available GEP data using ACSNI will lead to new insights into the molecular basis of a phenotype and its deregulation in disease.

It is well established that signaling pathways are coordinated by complex interdependences between chromatin structure, DNA modifications, RNA abundance and modifications, and protein abundance and modifications. While ACSNI includes the option to account for one additional piece of regulatory information as an integer variable, this is unlikely to model the complete interdependences. We plan to further develop the ACSNI software by expanding the utilization of other genomic information.

### Conclusion

ACSNI offers the cell and molecular biologist a flexible approach to infer potential pathway components that can be used to guide functional analysis in a target signaling network. The ACSNI package provides command-line functions (*ACSNI-run* and *ACSNI-derive*) for predicting the components of a signaling pathway from GEPs using prior knowledge. It also provides a function (*ACSNI-get*) to describe the correlation between the derived pathway subprocesses and biological traits. The ACSNI package includes a function (*ACSNI-split*) to process GEPs for bootstrap analysis. Users can install the Python package and its dependencies through the pip package installer (*pip install ACSNI*).

## Experimental procedures

### Resource availability

#### Lead contact

Further information and requests for the implementation and troubleshooting the ACSNI software should be directed to and will be fulfilled by the lead contact, Chinedu Anthony Anene (a.anene@qmul.ac.uk).

#### Materials availability

This study did not generate new materials.

#### Data and code availability

This paper analyzes existing, publicly available data. The ACSNI Python package is publicly available at GitHub, https://github.com/caanene1/ACSNI, and can be installed through the pip python installer. The code used to process and generate the figures reported in this paper is available at GitHub, https://github.com/caanene1/ACSNI. Any additional information required to reproduce this work is available on the package documentation at GitHub, https://github.com/caanene1/ACSNI, and from the lead contact.

##### Publicly available datasets and software

**Table:** 

REAGENT OR RESOURCE	SOURCE	IDENTIFIER
**Deposited data**		

TCGA ccRCC dataset	Cancer Genome Atlas Research Network, 2013^7^	https://portal.gdc.cancer.gov
GTEx expression datasets	Lonsdale et al., 2013^6^	https://gtexportal.org/home/v8_RNASeQ_v1.1.9
Validation ccRCC dataset	Yao et al., 2017^27^	https://www.ncbi.nlm.nih.gov/geo GSE102101
KFL6-ko data in ccRCC cells	Syafruddin et al., 2019^23^	https://www.ncbi.nlm.nih.gov/geo GSE115763
EPAS1-ko data in ccRCC cells	Zou et al., 2019^36^	https://www.ncbi.nlm.nih.gov/geo GSE115389
KFL6-ko data in blood cells	Adelman et al., 2019^26^	https://www.ncbi.nlm.nih.gov/geo GSE121560
EPAS1-ko data in endothelial cells	Yoo et al., 2015^37^	https://www.ncbi.nlm.nih.gov/geo GSE62974
mTOR-inhibition data in ccRCC cells	Kornakiewicz et al., 2018^28^	https://www.ncbi.nlm.nih.gov/geo GSE106819
HOTAIRM1-ko data in kidney cells	Hamilton et al., 2020^32^	https://www.ncbi.nlm.nih.gov/geo GSE136604
TF ChIP-Seq datasets	Oki et al., 2018^38^	https://chip-atlas.org
Cancer-cell dependency data	Tsherniak et al., 2017^29^	https://depmap.org/portal
Pathway interaction gene sets	Schaefer et al., 2009^17^	https://github.com/NCIP/pathway-interaction-database/tree/master/download
MSigDB v7.2	Liberzon et al., 2011^18^	https://www.gsea-msigdb.org/gsea/msigdb

**Software and algorithms**		

**ASCNI**	This paper	https://github.com/caanene1/ACSNI
Trimmomatic v0.39	Bolger et al., 2014^39^	http://www.usadellab.org/cms/?page=trimmomatic
HISAT2 v2.1.0	Pertea et al., 2016^40^	http://daehwankimlab.github.io/hisat2
HTSeq v0.11.1	Anders et al., 2015^41^	https://htseq.readthedocs.io/en/release_0.11.1
Python v3.8.6	PSF	https://www.python.org
R v4.0.3	CRAN	https://www.r-project.org

### Method details

#### ACSNI algorithm

We introduce ACSNI, an algorithm to infer the components of a biological pathway from bulk tissue expression profiles and prior knowledge of the pathway (Figure 1A). Let *P* denote the activity of the target pathway in a given biological sample and *K* (minimum of 4 genes) a curated set of genes associated with *P* based on current knowledge of their biological functions (gene set).^42^ We assume that *P* is organized into several small, highly connected subprocesses (*S*_*i–n*_) that combine hierarchically into larger units.^16^ A natural model is to have

(Equation 1.1) $$P\;=\;\sum_{i=1}^{n}S_{i},$$

where *S*_*i*_ is the contribution of the *i*^th^ subprocess to the pathway *P* and *n* is the total number of subprocesses.

While the hierarchical model is a faithful description of cell signaling pathways, a practical issue is that the number of subprocesses (*n*) is unknown a priori. Our best estimate of *n* is the current knowledge of the target biological pathway (*P*), as specified by the gene set *K*. To address this issue, we let *n* depend on the cardinality of the gene set *|K|*, defined as a percentage of *|K|* and estimated using a recent algorithm explicitly developed for autoencoders (described below).^43^ Alternatively, *n* can be specified by the user based on their knowledge of the target pathway.

For each *k* ∈ *K*, we have an estimate of its expression level across a cohort of *N* samples indexed by *j*. We let *V*_*k*_ be the scaled expression vector for gene *k* and *V*_*kj*_ is the expression of *k* in the *j*^th^ sample; determined by RNA-seq. Since genes act in multiple pathways and subprocesses,^44^ it is the additive change in expression within subprocesses that leads to the difference in pathway activity. Thus, we can model *s*_*n*_ in the sample cohort as a function of the expression of *k*:

(Equation 1.2a) $$S_{i}=\sum_{j=1}^{\left|K\right|}k_{j}w_{j}+b_{j},$$

where $b_{j}$ is a constant error term for the *j*^th^ sample, *w*_*j*_ is the influence of the *j*^th^ gene on the subprocess *S*_*i*_ and *k*_*j*_ is the expression value of *k* gene in the *j*^th^ sample. To account for additional prior knowledge, we include an optional weight adjustment to our model below:

(Equation 1.2b) $$S_{i}=\overset{\left|K\right|}{\underset{j=1}{\sum }}k_{j}w_{j}\cdot r_{j}+\;b_{j},$$

where *r* denotes the gene-level weights of the new information, such as TF activity, protein family, or kinase group.

### Unsupervised derivation of pathway subprocesses (*S*_*n*_)

ACSNI starts from the RNA expression values for the *K* genes and estimates the activities of the subprocesses across the samples. To this end, we implement a DNN for estimating *S*_*nj*_ through a pair of encoder and decoder layers (autoencoder):

(Equation 1.3) $$\bar{V_{k}}=d\left[f\left(V_{k}\right)\right],$$

where *f* (encoder) is a function of the expression to the pathway (*P*), *d* (decoder) is a function of the pathway to the expression, and ${\bar{V}}_{k}$is the reconstructed version of the expression vector. We train the network to find a solution to the optimization problem:

(Equation 1.4) $$\min{|}_{d,\;f}\left|\bar{V_{k}}-d\left[f\left(V_{k}\right)\right]\right||,$$

where ||. || is the l1 and l2 norms that disallow the identity map and forces the model to learn a sparse representation. The variation across the samples informs the compression and reconstruction of the expressions for all *k* ∈ *K*. We restrict feature selection to the removal of uninformative genes that have a median absolute deviation below a threshold (user-defined value >1, default 2.5). Figure S4A illustrates the mapping between the biological system, mathematical model and DNN layers.

To determine the optimal number of dimensions (i.e., the unknown *n* subprocesses in Equation 1.1), we implement a recent algorithm explicitly developed for estimating latent space dimension in autoencoders.^43^ Specifically, we determine the optimal latent space for each gene set expression profile by an iterative process, including:

1 Run the autoencoder above using 50% of the gene set's cardinality (50 × *|K|*/100) and extract the latent structure.
2 Apply the Bahadur and Paffenroth algorithm to this latent structure to estimate the optimal dimension.
3 Fix and run ACSNI with the estimated dimension.

In step 1, we initiated the search at 50% of the gene set based on simulation analysis that showed that although increasing the dimension of the latent space does not affect the ability to recover the expected signal (Figure S1D), it comes at the cost of increasing the FDRs (Figure S1E), which we want to minimize. Latent dimension below 50% of the gene set size appear to achieve the optimal balance between FDR and recovery of expected signals. Since our ultimate aim is to prioritize biological components without inflating false discovery, a small dimension is preferred.

Other dimension-reduction methods, including PCA and NMF, have been applied to gene expression datasets and could theoretically be used to estimate pathway subprocesses (*S*_*nj*_). Therefore, we also implement PCA and NMF, two linear dimension-reduction methods, into the ACSNI software, allowing users to cross-compare the results.

#### Estimation of subprocess-gene interaction scores

The neural network above minimizes the error between the input expression vector $V_{k}\;$and the reconstructed version (${\bar{V}}_{k}$). However, it is, not the values of ${\bar{V}}_{k}$ that are of interest to us but rather the subprocesses in each sample. Thus, after training the autoencoder, we extract the learned weights (*W* matrix) of the first hidden layer, which represents the reduction of the gene set expression profiles to latent structures. *W* has a dimension of *m × n* (*m =* the total number of samples and *n =* the number of estimated subprocesses) and is the activity of the subprocesses defined in Equation 1.2a or 1.2b.

For the rest of genes in the transcriptome, the strength of the linear relationship between their expression and the vector of *W* above corresponds to per-gene interaction between individual latent vectors and the gene. We model this interaction as

,(Equation 1.5) $$\;a=\;\beta S_{i}+\;\varepsilon \;,$$

where a is the expression profile of a gene in the rest of the transcriptome, *β* is the slope, *S*_*i*_ is the activity of *i*^th^ subprocess, and ε is the intercept, estimated automatically.

Next, for each run of Equation 1.5, we extract the R^2^ (here called interaction score *e*); this can be interpreted as the coefficient of determination for the proportion of the variance in that gene's expression across the samples that is predictable from each vector of the *W* matrix, defined as

(Equation 1.6) $$e=1-\;\frac{\sum {\left(a_{j}\;-\;\hat{a_{j}}\right)}^{2}}{\sum {\left(a_{j}-\;\bar{a}\right)}^{2}}\;,$$

where e is the interaction score, $a_{j}$ is the expression value for sample *j*, $\hat{a_{j}}$ is the predicted expression value for sample *j* and $\bar{a}$ is the mean expression value.

The Equations 1.5 and 1.6 were estimated using the Linear model function implemented in the sklearn Python package, using default parameters. We define the matrix of interactions scores (*e*) with genes in the row and subprocesses in the column as the (*N* matrix), which is used for prediction of unknown pathway components below.

Further, we extract the learned weights of the second hidden layer (*Code*), which represents the reduction of the subprocesses to the pathway. *Code* has a dimension of *m × n* (*m* is the number of estimated subprocesses and *n* is the number of estimated higher pathway units) and is the activity of the pathway defined in Equation 1.1. These data can be used for diagnostics and dimension plots.

#### Inference of tissue-specific components of the target pathway

Having estimated the multiple interactions between the subprocesses of the target pathway and genes in the rest of the transcriptome, we classify each subprocess independently and combine the predictions into a network. This process intuitively captures the signal contained in the gene set and thus represents a robust measure of the context-specific circuitry of the target pathway. Let *e*_*n*_ be the scores for the interaction between the *i*^th^ subprocess and the genes in the rest of the transcriptome (bound 0–1). e has a beta distribution parameterized by *α* and *β* (Figure S1F; see also analysis of probability distribution),

(Equation 1.7) $$\text{α}=\;\left(\frac{1-\text{μ}\;}{{\text{σ}}^{2}}-\;\frac{1}{\text{μ}}\right){\text{μ}}^{2}\;,$$

(Equation 1.8) $$\text{β}=\text{α}\left(\frac{1}{\text{μ}}-1\right),$$

where μis the mean interaction across the rest of the transcriptome with variance ${\text{σ}}^{2}$.

For a future experiment, it is the regularized incomplete beta function *I*_*j*_(α, β) that defines the impact of a subprocess on the *j* gene and in turn the pathway network. However, we find a cumulative distribution function to be an impractical network measure and therefore discretize it by defining the interactions to be *I*_*j*_(α, β) < 0.01 (*P* matrix). The cutoff can be interpreted as the probability threshold to reject the null hypothesis that such an extreme value is expected less than 1% of the time. Simulation analysis showed that the 0.01 cutoff reduces the FDR without affecting the ability to recover the expected signal compared with the traditional 0.05 for rejecting a null hypothesis (Figure S1B). The user can also adjust the cutoff depending on their aims.

Next, for each subprocess (Equation 1.2a or 1.2b), we extract the genes that reach the above threshold as the individual subprocess network. Finally, we represent our global network for the pathway (Equation 1.1) as the union of all the genes predicted across the subprocesses, thus a global network across all subprocesses. Since the neural network optimization process is stochastic, we implement an ensemble learning and combine predictions using summary statistics. Here, we count the number of times a gene is predicted across the ensemble and retain genes predicted in at least two models. We suggest using at least an ensemble of three models to increase the stability of the predictions.

#### Mapping subprocess activity to external variable

Here, we define a subprocess significance measure as a function *SS* that assigns a non-negative value (0–1) to each subprocess; the higher is *SS*_*w*_, the more biologically significant is subprocess *w* for the given variable. We derive *SS* using logistic regression for categorical variable or simple linear regression for numerical variables, implemented as the ACSNI-get module (see code at https://github.com/caanene1/ACSNI).

#### Architecture and hyperparameters

The neural network used in ACSNI consists of two fully connected layers and a mirror image of this neural network as a decoder (Figure S4A shows the map between the biology, equations, and the network layers). We used *x* neurons in the first layer, and (*x*/2) neurons in the second layer of the network. *x* is automatically determined, as described in estimation of subprocess-gene interaction scores, or is user defined based on prior knowledge. During the subprocess-gene interaction analysis, we extracted the learned weights of the first layer (autoencoder) or the loading of the linear dimension-reduction controls (PCA and NMF controls) and used these to predict gene expression values. We used RMSprop optimizer with learning rate of 1 × 10^−3^ for training. Activities of the neurons were normalized using layer normalization that calculates the normalization statistics for all units in the same layer. The RELU function, defined as RELU(*x*) = max(0, *x*), was used as a non-linear activation in the two layers. In the mirror image, the Sigmoid function, defined as Sigmoid(*x*) = 1/1 + *e*^−*x*^, was used as the activation function. We train the model for 3,000 epochs and configure the checkpoint to save at the end of every epoch, if it is the best model seen (i.e., the lowest mean squared error in the ACSNI objective function [1.4]). Regularizers l1 and l2 in the ACSNI objective function were set to 1 × 10^−16^ and 1 × 10^−9^, respectively. ACSNI is implemented in Python 3 and relies on the tensorflow, sklearn, numpy, and pandas packages.

#### Requirements

The validity of the approach is related to the quality of the starting gene set; the higher the quality of the sources that link the genes in a pathway, the better the subprocess estimates. The changeable and partially arbitrary nature of gene annotations should be considered before treating gene sets as units of biological processes. It is a further requirement that the sample cohort should minimally contain 50 samples and come from the same type of tissue.

#### *De novo* generation of gene sets (*ACSNI-derive*)

The requirement for a pre-annotated gene set is a challenge because many biological processes remain completely unknown. For example, few gene sets are available for the pathways of non-coding RNAs. To address this problem, we develop the *ACSNI-derive* tool for *de novo* creation of a gene set given a single gene. First, the expression of the gene of interest is correlated with the expression of other genes (this can also be set for a specific biotype only, for example, lncRNA, microRNAs, or protein coding). The topmost correlated targets are then selected to construct a search space and negative controls (top 50 with r > 0.6 and r < −0.6). We search this space using multiple iterations of ACSNI and extract genes predicted in more than 60% of the iterations in the presence of the target as the functional gene set (see code at https://github.com/caanene1/ACSNI).

#### Null models

For the evaluation of randomness in the ACSNI predictions, we randomly shuffled the gene expression values of each sample. This process generates random expression profiles without altering the samples' library sizes. We then apply the ACSNI model as described before under the assumption that the predictions must be false positives, originating from an uninformative pattern extraction in the *W* matrix and interaction prediction (*P* matrix). This process is included in the ACSNI software and generated automatically.

#### Datasets for simulation studies

To evaluate how well ACSNI reconstructs a known signal and its FDR, we created pseudo-simulated RNA-seq data. We did not use purely synthetic data because we wanted to ensure that our simulation reflects the expected results from real RNA-seq datasets. Specifically, we extracted the RNA expression values from the GTEx project (here called expression data) and 50 gene sets from the MSigDB database.^18^ The expression data have genes in rows and samples in columns (here called rows and columns, respectively). The gene sets are sets of gene names (we refer to their expression profiles as gene set signal). Next, we generated a pair of 50 simulated expression data and gene set in three independent data transformations:

1 For a given gene set, split the expression data into two groups: (1) expression of genes in the gene set (data 1) and (2) expression of the rest of the genes (data 2).
2 .Individually shuffle the column values of data 2, such that the row values are randomly assigned without altering the original column sums (this is the random signal).
3 For each row in data 1, individually reverse the ordering of the values (right to left), whereby the last column gets the expression in the first column and the first column gets the expression in the last column (this is the expected signal). This signal is expected because ACSNI is invariant to the direction and maintains the order of the columns.
4 Create ten versions of data 1 with different log-normal noise levels (5%, 6%, 7%, 8%, 9%, 10%, 20%, 30%, 40%, and 50%) randomly added to each value (this is the set of expected-noise signals). This signal can be interpreted as the technical noise caused by RNA-seq and alignment errors. Each of the ten versions has one level of noise and independent of the other.
5 Finally, for the given gene set merge the transformed datasets from steps 2 to 5 with data 1 to form one expression matrix (this is the pseudo-simulated dataset with known signals). Output the row names of data 1 as the new gene set, creating expression and gene set pairs.

Note that these datasets were analyzed with ASCNI as pairs because each expected signal is specific to a given gene set. We provide details of the ACSNI parameters used to analyze the simulated datasets in the results section.

### Quantification and statistical analysis

#### Comparison of methods for estimating pathway activity

Other methods are available for estimating sample-wise pathway activity scores that can be used to infer pathway components comparable with global ACSNI predictions. To benchmark the global predictions, we compared performance on the validation datasets (genetic perturbations or DNA occupancy data) with four alternative approaches: GSVA, PLAGE, ssGSEA, and *Z* score.^12^, ^13^, ^14^, ^15^ These methods estimate a single activity score for each gene set and individual sample. In the presented mTOR and ATF2 examples, we used the version of GSVA, PLAGE, ssGSEA, and *Z* score implemented in the GSVA R package to estimate pathway activity for each sample. We then correlated the pathway activities from each method with the expression of each gene across the samples. The gene-wise correlation coefficients were classified at >0.4 and < −0.4 to predict pathway genes (see code at https://github.com/caanene1/ACSNI). We did not apply the ACSNI custom classification method to correlation coefficients because our approach required a beta distribution, and the correlation coefficients have a different distribution (Figure S4B).

For the single gene example (HOTAIRM1 annotation using *ACSNI-derive* module), we compared the ACSNI prediction with the pairwise correlation analysis and differential expression analysis between discretized expression groups (HOTAIRM1-high: >median versus HOTAIRM1-low: <median). For correlation analysis we used the default pearsonr and for differential expression, ttest_ind implemented in the SciPy Python package. Genes were classified as pathway components at correlation coefficient of >0.6 and <−0.6 or two-tailed adjusted p values of <0.05 (see code at https://github.com/caanene1/ACSNI).

We estimated the subprocesses activity encoded in the gene set expression profiles by PCA and NMF dimension reduction to compare ACSNI-step 1 with linear dimension reduction. We used the default PCA and NMF functions implemented in the sklearn Python package. The optimal number of dimensions was determined by the number of components required to explain 95% variation in the expression profiles of the gene set. The loading for each component was extracted (i.e., subprocess activity per sample) and used in the rest of the ACSNI calculations.

For all methods, the input data were either RSEM or transcripts per million (TPM), which are the preferred data transformations and are also used in ACSNI.

#### RNA-seq analysis

For GEO RNA-seq datasets, the raw reads were filtered to remove the adaptors and the low-quality reads (Q < 20) using Trimmomatic.^39^ Filtered reads were aligned to the human reference genome GRCh38/hg38 assembly using HISAT2 (v2.1.0) in default settings.^40^ The counts in different genomic features were then generated using HTSeq (v0.11.1)^41^ on human GRCh38 reference annotation (GENCODE Release 32). The expression levels were normalized by TPM. Differential expression analyses between two groups were performed using the limma R package. The DE genes were defined at adjusted p value of <0.05.

#### Gene ontology analysis

The R ClusterProfiler and the human Bioconductor annotation database (org.Hs.eg.db) were used to investigate the biological processes associated with the predicted components of the studied pathway. Ontologies were filtered for redundancies by semantic similarity analysis^45^ and considered significant at *q* value <0.01 or otherwise stated in the figure legends.

#### Randomization test

We utilized a non-parametric randomization test to evaluate the significance of the overlap between the genes predicted by ACSNI and genes in a validation signal, or between two ACSNI predictions. For the comparator variable, we randomly reassigned the labels and calculated the overlap statistic with the target labels. We repeated this process 10,000 times and counted the number of times the overlap statistic was greater than the observed statistic. We then estimated the empirical probability (empirical p) as the count divided by the total number of randomizations (i.e., 10,000). The R implementation of the randomization test analysis is available at https://github.com/caanene1/ACSNI.

#### Analysis of probability distribution

The R fitdistrplus and logspline packages were used to investigate the interaction scores' distribution (Equation 1.6). We computed descriptive parameters of the score vector and visualized the skewness-kurtosis plot to assess the possible distribution. We then fitted the set of possible distributions to the scores and compared their density, quantiles, and probabilities with theoretical values. We selected the best distribution for the data based on the lowest Akaike information criterion (AIC) across all possible distributions (i.e., we used distribution with the lowest AIC). The R script for distribution analysis is available at https://github.com/caanene1/ACSNI.

## Supplemental information

Document S1. Figures S1–S4

Document S2. Article plus supplemental information
