# Supplementary material for: ACSNI: An unsupervised machine-learning tool for prediction of tissue-specific pathway components using gene expression profiles
Source: Patterns (N Y). 2021 Jun 11;2(6):100270. doi: 10.1016/j.patter.2021.100270 (PMC8212143; doi:10.1016/j.patter.2021.100270)
Supplement: Document S1. Figures S1–S4 [file mmc1.pdf]

Patterns, Volume *2*

Supplemental information

ACSNI: An unsupervised machine-learning tool for prediction of tissue-specific pathway components using gene expression profiles

Chinedu Anthony Anene, Faraz Khan, Findlay Bewicke-Copley, Eleni Maniati, and Jun Wang

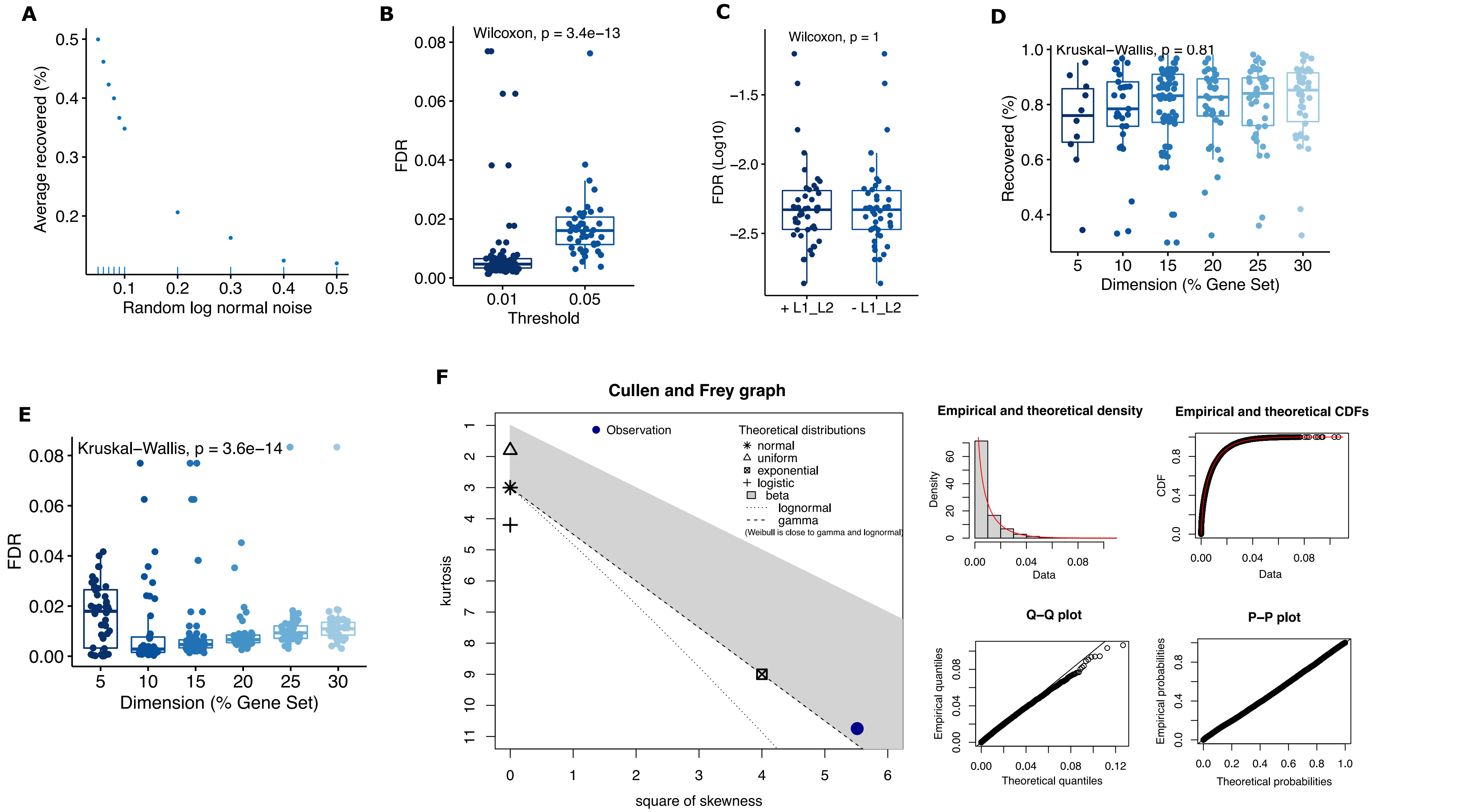

Figure S1: Simulations studies reveals optimal parameters for ACSNI.

A) Dot plot depicting the effect of random noise (log normal) on the average percentage of gene set signal recovered from the analysis of 50 gene sets against simulated expression profiles (see Methods). **B-C)** Box plot comparing the level of FDR across **B** alpha thresholds and **C** with or without L1_L2 regularisation in the simulated expression profiles. **D-E)** Box plots depicting the effect of latent space dimension (percentage of gene set size) on the **D** percentage of gene set signal recovered and **E** false discovery rate in simulated expression profiles analysed with 50 random gene sets. **F)** Representative Cullen and Frey graph of the kurtosis and square of skewness of ACSNI estimated subprocess interaction scores (e) **(left panel)**. The plot indicates the expected location of some probability distributions. **(right panel)** Plots of empirical and theoretical density, cumulative density functions, quantiles (Q) and probabilities after fitting e to a beta distribution.

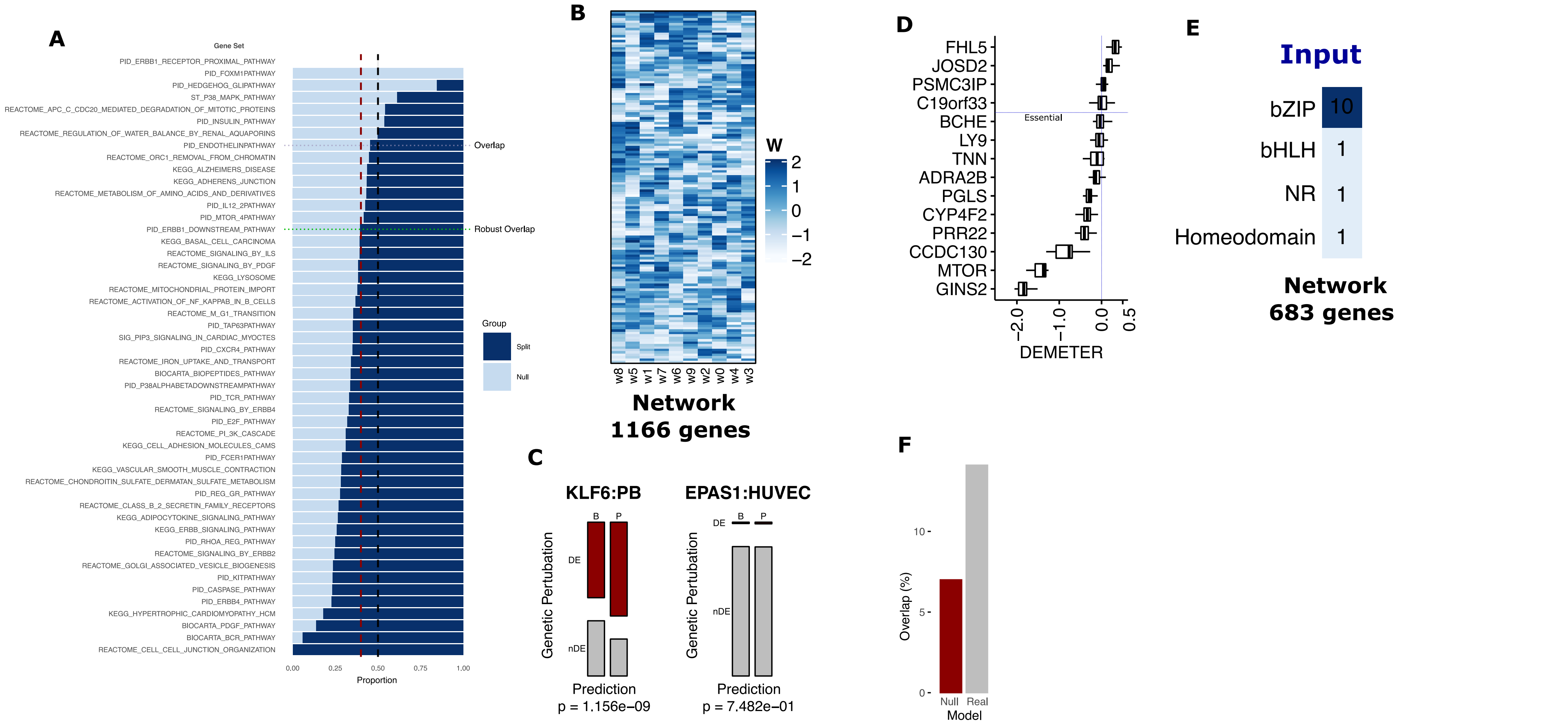

Figure S2: ACSNI predictions are robustly validated and not random.

A) Similarity of predicted genes for 50 curated gene sets between two independent split (Split) or with one shuffled split (null) of the GTEx lung tissues expression data. The black and red broken lines represent 50% and 75% of the real similarity relative to the null, respectively. **B)** Heat map of the resulting W matrix, facilitating the inference of the 1166 genes strongly associated with mTOR signalling subprocesses in TCGA KIRC. **C)** Association of ACSNI predicted mTOR signalling genes (TCGA) to DE genes in KLF6 and EPAS1 knock-out in non-ccRCC cells divided into cell types (KLF6: PB = GSE121560, EPAS1: HUVEC = GSE62974). **D)** Distribution of DEMETER2 (dependency scores) for mTOR subprocess w8-associated genes from TCGA KIRC in ccRCC cell lines analysed in the DepMap project (https://depmap.org/portal/). **E)** Frequency of DNA binding domains associated with the TFs in the ATF2 gene set **(Upper panel)** and the number of genes predicted in the artery aorta expression data **(Lower panel)**. **F)** Bar plot comparing overlap with DE genes from HOTAIRM1-KO with ACSNI predicted HOTAIRM1 genes between the real kidney expression and shuffled expression data (Null model).

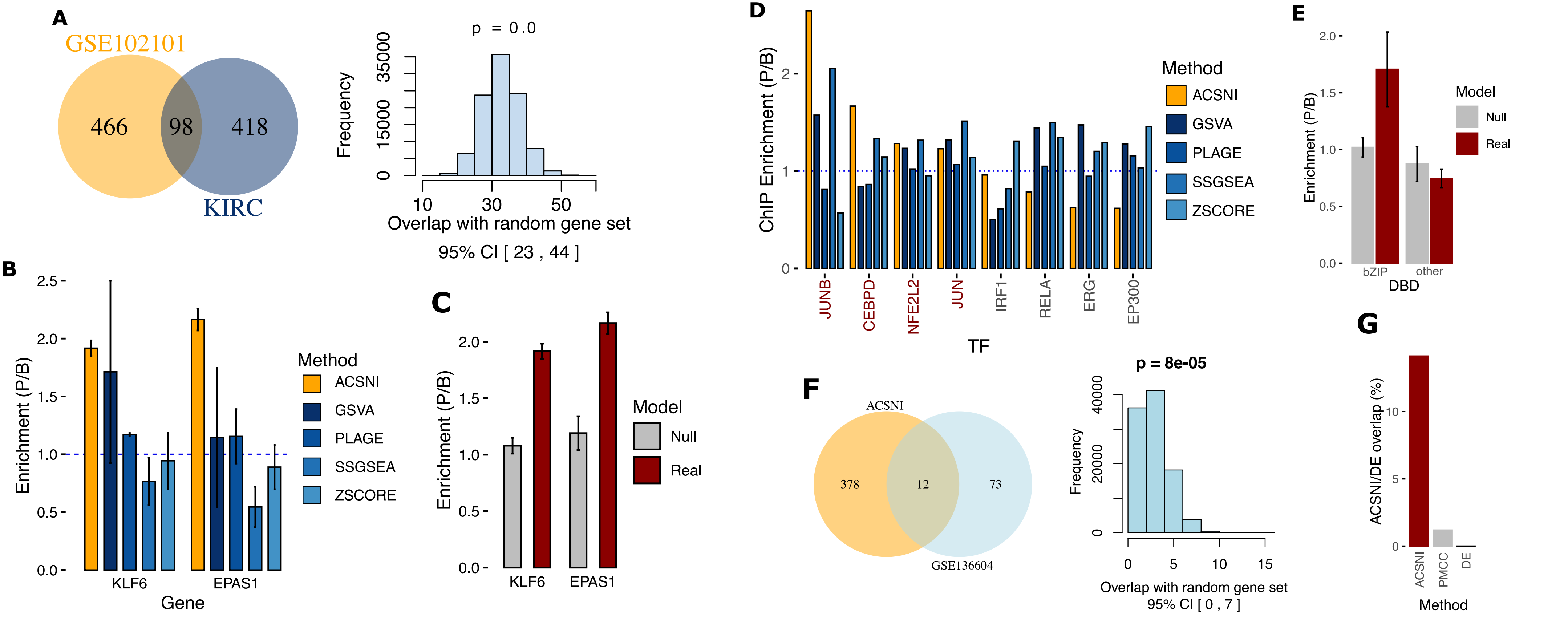

Figure S3: ACSNI is superior to existing approaches.

A) Venn diagram showing the overlap between ACSNI predicted mTOR signalling genes from ccRCC TCGA (KIRC, n = 146) and GEO (GSE102101, n = 22) datasets for genes measured in both datasets **(left panel)**. Frequency of the number of overlaps between ACSNI predicted mTOR signalling genes from ccRCC TCGA and random genes (size = 1166, n = 100,000), with empirical probability for an overlap >= 98 **(right panel)** is indicated. **B)** Bar plot comparing the ratio of the proportion of predicted mTOR signalling genes and background genes that were also differentially expressed in KLF6 or EPAS1 knock-out in 786 cell lines across different methods. Within the plot, error bars were calculated from the ratio in KIRC and GSE102101 datasets. GSVA, PLAGE, SSGSEA and ZSCORE represents the methods (see Methods) used to generate pathway activity scores before correlation analysis (DE). Correlation coefficients (± 0.4) was used to classify pathway components. **C)** Bar plot comparing enrichment of DE genes from KLF6-KO and EPAS1-KO (red) in ACSNI predicted mTOR signalling genes between the real expression and shuffled expression data (Null model). Error bars were calculated from the ratio in KIRC and GSE102101 datasets. **D)** Bar plot comparing the ratio of transcription factor (TF) ChIP density at the promoter regions (+/- 1k TSS) of the ATF2 signalling genes relative to background genes in primary endothelial cells across different methods, as described in **B**.
E) Bar plot comparing ratio of TF binding for bZIP and other TFs in ACSNI predicted ATF2 signalling genes between the real artery aorta expression and shuffled expression data (Null model). Error bars were calculated from the enrichment in four bZIP TFs (JUN, JUNB, CEBPD and NFE2L2) or four other TFs (IRF1, RELA, ERG AND EP300). **F)** Vein diagram showing the overlap between ACSNI predicted HOTAIRM1 genes in kidney tissues and differentially expressed (DE) genes in HOTAIRM1-KO data (GSE136604), **(left panel)**. Frequency of the number of overlaps between ACSNI predicted HOTAIRM1 genes and random genes (size = 683, n = 100,000) from kidney tissues **(right panel)**, with empirical probability for an overlap > 12. **G)** Bar plot comparing the overlap between the predicted HOTAIRM1genes and DE genes in HOTAIRM1 knock-out across different methods direct correlation (PMCC) or discretised expression groups with DE test.

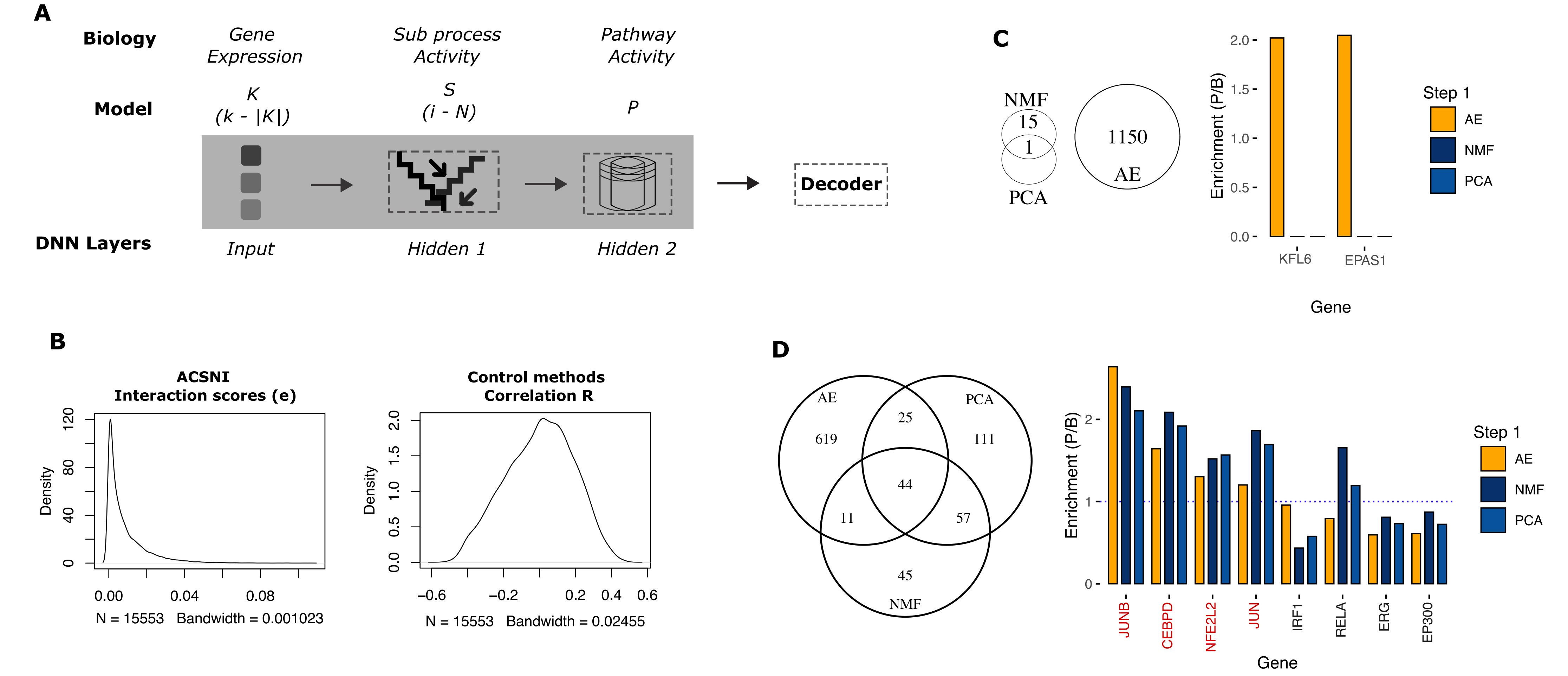

Figure S4: Map of biological pathways to the ACSNI algorithm.

A) The expression profile of the gene set (K, indexed by k) represents the input layer, subsets of K genes organise into sub process activity (S, indexed by i) represented as the hidden layer 1, which in turn organise to form the pathway activity (P) represented as the hidden layer 2. **B)** Representative density plot of the distribution of ACSNI derived interactions scores **(left panel)** compared to the distribution of correlation coefficients **(right panel)**. **C)** Venn diagram showing the overlap between ACSNI predicted mTOR signalling genes from ccRCC TCGA (KIRC, n = 146) using autoencoder (AE), principal component analysis (PCA) or non-negative factorisation (NMF) to estimate subprocess activity in step 1**(left panel)**. Bar plot comparing the ratio of the proportion of predicted mTOR signalling genes and background genes that were also differentially expressed in KLF6 or EPAS1 knock-out in 786 cell lines across different methods of methods of step 1 **(right panel)**. **D)** Venn diagram showing the overlap between ACSNI predicted ATF2 signalling genes in GTEx artery aorta (n = 432) using autoencoder (AE), principal component analysis (PCA) or non-negative factorisation (NMF) to estimate subprocess activity in step 1 **(left panel)**. Bar plot comparing the ratio of transcription factor (TF) ChIP density at the promoter regions (+/- 1k TSS) of the ACSNI predicted ATF2 signalling genes relative to background genes in artery aorta lines across different methods of step 1 **(right panel)**.
